# The financial toll of cancer: uncovering the links between financial toxicity and symptom burden

**DOI:** 10.1093/oncolo/oyaf131

**Published:** 2025-06-26

**Authors:** Marco Filetti, Pasquale Lombardi, Gabriella Gentile, Paolo Toccaceli, Guglielmo Fumi, Denise Vacca, Emilia Colpani, Giulio Ravoni, Federica Mazzuca, Gennaro Daniele, Giampiero Porzio, Eduardo Bruera, Raffaele Giusti

**Affiliations:** Phase 1 Unit, Fondazione Policlinico Universitario A. Gemelli, IRCCS, 00168 Rome, Italy; Phase 1 Unit, Fondazione Policlinico Universitario A. Gemelli, IRCCS, 00168 Rome, Italy; Division of Cancer, Department of Surgery and Cancer, Imperial College London, Hammersmith Hospital, London W12 0HS, UK; Medical Oncology Unit B, Department of Radiology, Oncology and Pathology, Policlinico Umberto I, Sapienza University of Rome, 00161 Rome, Italy; Hospice Care Unit, USL Umbria 1, 06126 Perugia, Italy; Azienda Ospedaliera S. Maria S.C. Oncologia, 05100 Terni, Italy; Palliative Care Unit, Ospedale Sirai, Carbonia, ASSL 09013 Carbonia, Italy; Tuscany Tumors Association, Home Care Service, 50132 Florence, Italy; Tuscany Tumors Association, Home Care Service, 50132 Florence, Italy; Department of Clinical and Molecular Medicine, Sapienza University, Medical Oncology Unit, Azienda Ospedaliera, 00189 Rome, Italy; Phase 1 Unit, Fondazione Policlinico Universitario A. Gemelli, IRCCS, 00168 Rome, Italy; Tuscany Tumors Association, Home Care Service, 50132 Florence, Italy; Department of Palliative, Rehabilitation, and Integrative Medicine, The University of Texas MD Anderson Cancer Center, Houston, TX 77030, USA; Medical Oncology Unit, Azienda Ospedaliera Universitaria Sant’Andrea, 00189 Rome, Italy

**Keywords:** financial toxicity, out-of-pocket costs, symptom burden, healthcare economics

## Abstract

**Purpose:**

This prospective observational study investigated the relationship between financial toxicity, symptom burden, disease characteristics and out-of-pocket (OOP) medication use in patients with cancer across 5 Italian institutions.

**Methods:**

A cross-sectional survey assessed financial toxicity, symptom burden and OOP costs sustained by patients with cancer using validated questionnaires (COST, PERSONS). Associations between financial toxicity (COST score), symptom burden (PERSONS score) and clinical variables were explored.

**Results:**

Among 211 respondents, mean COST score was 23 (SD 9.1) and 128 (60%) scored < 26, indicating financial toxicity. Symptom burden measured by the PERSONS score was associated with a higher OOP medications use (*r* = 0.449, *P* < .001) and inversely related to financial toxicity (*r* = −0.431; *P* < .001). In our study population, median number of OOP drug was 2 (IQR 1-2), most commonly analgesics and laxatives. There was a significantly worse COST score among patients using OOP analgesic: 19.8 (SD 9.5) vs 25.6 (SD 7.9), *P* < .001 and laxatives: 19.2 (SD 8.2) vs 24.4 [(SD 9.1), *P* < .00]. Overall, OOP drugs were linked to higher symptom burden (*P* < .001) and financial toxicity (*P* < .001).

**Conclusions:**

The severity of symptoms and their management with OOP medications was significantly associated with patients’ financial toxicity. These findings highlight the importance of integrating financial toxicity assessment into routine cancer care to optimize patient outcomes and well-being.

**Implications for Practice**

- Financial toxicity significantly impacts cancer patients even in universal healthcare systems.
- Implementing proactive financial counseling and developing policies to cover supportive care medications could improve treatment adherence and outcomes.
- Healthcare providers should consider the financial impact of symptom management strategies and work toward integrated approaches that address both clinical and economic dimensions of cancer care to reduce the cyclical relationship between symptom burden and financial distress.

## Introduction

Cancer represents a significant public health challenge, not only due to its physical and psychosocial consequences but also due to the substantial financial burden it imposes on patients. This financial burden, termed “financial toxicity,” encompasses the direct costs of treatment (eg, medications, procedures, admissions) and indirect costs (eg, lost income, transportation) associated with cancer care.

The financial implications of cancer treatment are a pressing global concern. While studies from the United States have extensively documented the prevalence of financial toxicity, highlighting outcomes such as increased bankruptcy rates and asset depletion,^1,2^ evidence suggests that financial toxicity is also pervasive in public healthcare systems worldwide.^3-5^ These findings underscore the urgent need for standardized measures and effective interventions to mitigate the financial strain associated with cancer care.

Financial toxicity can significantly impact patients’ decisions and lead to suboptimal cancer outcomes.^6^ It is a major source of distress for patients with cancer^7,8^ often contributing to reduced treatment adherence, poorer clinical outcomes, diminished quality of life (QoL), and lower patient satisfaction.^9-11^ Moreover, financial toxicity can exacerbate symptom burden amplifying physical and emotional distress and complicating symptom management.^12^

Out-of-pocket (OOP) costs represent a critical component of financial toxicity in patients with cancer. High OOP expenses, including copayments for essential medications, can adversely affect treatment adherence, potentially influencing disease outcomes.^13^ Financial concerns can also force patients to delay or forgo essential treatments, medications, and supportive care services, worsening symptom control and overall outcomes.^1,14^

Understanding the interplay between financial toxicity, symptom burden, and demographic factors is essential for developing effective supportive care interventions. A comprehensive approach to cancer care, extended beyond medical management to encompass the socioeconomic well-being of patients and their families. Recognizing and mitigating financial toxicity is essential for delivering holistic and patient-centered care.

To investigate the determinants of financial toxicity within the Italian healthcare system, this study aimed to determine the extent of OOP medication use among patients with cancer and describe the association between financial toxicities, symptomatology, demographic characteristics, and OOP medication use.

## Methods

### Setting and participants

Between January and December 2022, a cross-sectional study was conducted among patients with cancer enrolled from both outpatient and home care settings. The patients were recruited across 5 Italian Institutions. The study protocol was reviewed and approved by the ethics research committee at Sapienza University of Rome, Italy (approval number: Prot. n. 79 SA/2022 Ref. CE 6691/2022). The study also adheres to the principles of the Declaration of Helsinki. Patients provided written informed consent prior to participating in the study. The STROBE guidelines (see Supplementary 1) for observational studies were consulted during the design and reporting of this cross-sectional study.^15^

Respondents were eligible for participation if they were over 18 years of age and a diagnosis of advanced cancer (locally advanced or metastatic). In the outpatient group, all patients underwent a concomitant antineoplastic treatment (intravenous and/or oral), and, in the home care group, patients who were “out of treatment” were also enrolled. Patients were excluded if they were unable to provide consent or complete the questionnaire due to physical or cognitive limitations.

### Outcome variables

The study collected patient data including sociodemographic characteristics (age, sex, education level, employment status, income source), clinical information (cancer type, performance status, disease history), symptom burden, financial distress, and OOP costs.

### Questionnaire and variable characteristics

The data collection methodology consisted of an individual questionnaire administered by a professional interviewer. The questionnaire consists of 3 domains: sociodemographic and clinical characteristics of the patient with cancer, financial toxicity measure, and symptoms related to the disease.

Sociodemographic characteristics were collected as follows: Age was grouped into the following categories: ≤70 or >70. Education was classified as low, medium, and high level (ie, lower level includes no qualification and elementary school diploma, the medium level includes junior school and high school, and higher level includes degree and postdegree). Disease phase was dichotomized by grouping patients into 2 categories: active antineoplastic treatment and supportive/pain therapy only. For patients with an active antineoplastic program, therapies were indicated if simultaneous care was active, whereas for patients in supportive/pain therapy only, it was indicated if they were provided in a home or hospital setting. Cancer site was grouped into 7 categories, considering that the interviewed patient or the corresponding caregiver identified the cancer site (ie, Gastrointestinal, Thoracic, Breast, Gynecological, Prostate, Melanoma, and Miscellaneous cancers).

Financial toxicity among the patients with cancer was assessed by collecting the OOP costs related to cancer and using the Comprehensive Score for Financial Toxicity-Functional Assessment of Chronic Illness Therapy (COST-FACIT) questionnaire.

The COST—FACIT questionnaire consists of 12 questions (Supplementary 2). It was developed by De Souza et al.^16^ and was translated in Italian and validated to assess the degree of financial toxicity experienced by patients with cancer.^17^ The COST questionnaire has 12 items that have been officially coded sequentially from FT1 to FT12. Items 2, 3, 4, 5, 8, 9, and 10 of the questionnaires are reverse scored. The responses vary from 0 (not at all), 1 (a little bit), 2 (somewhat), 3 (quite a bit), and 4 (very much). The score ranges between 0 and 44, and a higher score implies lower financial toxicity. A cutoff of 26 was used as a threshold of COST to identify financial toxicity while^18,19^ scores of 14 or below were classified as “severe” financial toxicity, in line with previous studies.^19,20^

Out-of-pocket costs were identified by asking patients or caregivers about the direct expenses they sustained, which were not covered or only partially covered by the National Healthcare System (NHS), for the following health-related goods and services: anorexia and cachexia, pain, diarrhea, constipation, fatigue, nausea and vomiting drugs; with free text possibilities for other drugs or services.

Symptoms related to disease were assessed using PERSONS score. The PERSONS score is an easy and validated tool for symptom assessment and includes the following items: pain, eating (loss of appetite), rehabilitation (asthenia), sleep (sleep disorders), O2 (dyspnea, cough), nausea/emesis, and suffering (anxiety/ depression). Each item is rated on a numeric scale between 0 (no burden) and 10 (worst imaginable burden). All 7 points are summed, resulting in an overall score between 0 and 70 (Supplementary 3).^21^

### Data analysis

Descriptive statistics were used to summarize demographic and clinical characteristics. Continuous variables were reported as means and standard deviations, while categorical variables were presented as frequencies and percentages.

The relationship between the total PERSONS score and COST was investigated using Pearson correlation coefficient (*r*) with 95% CIs. The Pearson correlation coefficients (*r*) were interpreted as follows: ≤0.19, very weak correlation; 0.20-0.39, weak correlation; 0.40-0.69, moderate correlation; 0.70-0.89, strong correlation; and 0.90-1.00, very strong correlation. Differences in mean scores between subgroups were evaluated using both parametric (eg, *t*-tests, linear regression) and nonparametric tests (eg, Mann-Whitney *U*, Kruskal–Wallis) were employed as appropriate. A *P*-value < .05 was considered statistically significant.

All data were tabulated and coded using Microsoft Excel (v 2405). Statistical analyses were performed with SPSS V.29.0 (SPSS).

## Results

A total of 211 patients were recruited and completed all the item of the proposed questionnaires. Patient demographics, shown in Table 1, included the following: 43% (*n* = 91) of patients were 70 years age or older, 61.2% (*n* = 130) were retired and 27% (*n* = 57) were still working at the time of the survey. Educational attainment was diverse, with 8% having a high level of education, 39.3% a medium level, and 53.6% a low level. Gastrointestinal (38%) and lung (26%) were the most prevalent tumors within this cohort. Most responders exhibit an ECOG performance status score of ≤2 (71%), indicating good performance status. Notably, nearly half of patients (47.4%) received supportive and palliative care as their primary treatment. In the entire cohort of responders, 60.7% (*n* = 128) had a COST score <26, indicating they were experiencing financial toxicity. Of the 128 patients experiencing financial toxicity, 25% (*n* = 32; 15.2% of the total sample) scored <14, suggesting severe financial toxicity (Table 1).

**Table 1.** Characteristics of participating patients.

Variable	Number of patients (%)
**Sex**	
Male	101 (47.9%)
Female	110 (52.1%)
**ECOG**	
0-2	149 (70.6%)
3-4	62 (29.4%)
**Age class**	
≤70	120 (56.9%)
>70	91 (43.1%)
**Education level**	
Low	111 (53.6%)
Medium	83 (39.3%)
High	17 (8.1%)
**Cancer site**	
Colon-rectum	81 (38.4%)
Lung	55 (26.1%)
Breast	26 (12.3%)
Melanoma	4 (1.9%)
Gynaecological	10 (4.7%)
Prostate	8 (3.8%)
Others	27 (12.8%)
**Disease phase**	
Active treatment	111 (52.6%)
Only symptomatic treatment	100 (47.4%)
**Employment status**	
Employed	57 (27%)
Retired	130 (61.2%)
Unemployed	24 (11.4%)
**COST**	
Scored ≥ 26	83 (29.3%)
Scored < 26	128 (60.7%)
Scored ≤ 14	32 (15.2%)^a^
**Supportive care**	
No	107 (50.7%)
Among patients in active treatment	42 (19.9%)
Among patients in symptomatic treatment only	62 (29.4%)

^a^Patients with COST scored ≤ 14 are included in the group of patients scored <26 and represent 15.2% of the entire sample size.

We then examined patients self-administering OOP medications to manage symptoms. Among the 211 patients, 48 (22.7%) did not utilize any symptomatic medications, while the remaining 163 (77.3%) employed at least one drug with median OOP medications use in the entire population of 2 drugs (IQR 1-2). Analgesics (45%) and laxatives (28%) were the most common medication categories (Table S1).

Mean score for COST and PERSONS in the entire population were 23 (SD 9.1) and 27.5 (SD 14.1), respectively (Table 2 and Table 3). As expected, mean COST score did not significantly differ between patients aggregated for sex, performance status and age classes, and treatment phases. Nevertheless, patients with low and medium level of education exhibited lower mean COST score compared to patients with high education level (*P* .007). Similarly, unemployed patients had lower COST scores compared to those who were retired or employed at the time of the survey (*P* .019) (Table 2).

**Table 2.** Differences in COST.

Variable	Mean (SD)	*P*-value
**Sex**		
Male	22.3 (SD 8.2)	.302
Female	23.62 (SD 9.9)	
**ECOG**		
0-2	23.3 (SD 9.6)	.502
3-4	22.3 (SD 7.8)	
**Age class**		
≤70	22.3 (SD 9.3)	.194
>70	23.9 (SD 8.8)	
**Education level**		
Low	20.9 (SD8.4)	<.001^a^ <.001^a^
Medium	23.5 (SD 8.9)	
High	33.6 (SD 6.8)	
**Disease phase**		
Active treatment	22.5 (SD 8)	.456
Only symptomatic treatment	23.4 (SD 10)	
**Employment status**		
Employed	23.7 (SD 9.3)	.019^b^ .007^b^
Retired	23.7 (SD 8.7)	
Unemployed	17.7 (SD 9.4)	

^a^Compared with High education level group.

^b^Compared to unemployed.

**Table 3.** Differences in PERSONS

Variable	Mean (SD)	*P*-value
**Sex**		
Male	28.9 (SD 14.2)	.149
Female	26.1 (SD 13.9)	
**ECOG**		
0-2	24.2 (SD 13.2)	<.001
3-4	35.3 (SD 13.1)	
**Age class**		
≤70	25.9 (SD 13.9)	.059
>70	29.6 (SD 14.3)	
**Education level**		
Low	30.7 (SD14.2)	.001^a^ .118^a^
Medium	25.2 (SD 13.2)	
High	18 (SD 11.2)	
**Disease phase**		
Active treatment	24.4 (SD 13.4)	<.001
Only symptomatic treatment	31 (SD 14.1)	
**Employment status**		
Employed	28.1 (SD 16.1)	1^b^ .835^b^
Retired	26.5 (SD 12.4)	
Unemployed	31.2 (SD 17.0)	

^a^Compared with High education level group.

^b^Compared to unemployed.

In addition, PERSONS score did not vary by sex, age group and employment status. However, patients with worse performance status (*P* < .001), those receiving palliative care (*P* < .001) and those with low education levels compared to high education level (*P* < .001) had a higher symptoms burden in our analysis (Table 3).

Patients procuring medications OOP for pain and constipation exhibited a lower average COST score, indicating a higher risk of financial toxicity among these patients (Table 4).

**Table 4.** Differences in COST score in patient that used OOP medications.

Variable	Mean (SD)	*P*-value
OOP medication		
Pain relief		
Yes	19.8 (SD 9.5)	<.001
No	25.6 (SD 7.9)	
AntiDiarrheal		
Yes	23.1 (SD 5.4)	.940
No	22.9 (SD 8.1)	
Constipation		
Yes	19.2 (SD 8.2)	<.001
No	24.4 (SD 9.1)	
Anti-emetics		
Yes	21.4 (SD 9.5)	.252
No	23.3 (SD 9.5)	
Anorexia and Cachexia		
Yes	20.65 (SD 7.4)	.228
No	23.24 (SD 9.3)	
Cancer-Associated Fatigue		
Yes	20.7 (SD 10.2)	.061
No	23.6 (SD 8.7)	
Mood and anxiety		
Yes	20.5 (SD 9.4)	.246
No	23.2 (SD 9.1)	
Food supplements		
Yes	20.1 (SD 9.3)	.066
No	23.5 (SD 9.0)	
Mobility aids, nursing care beds, and others assistive devices		
Yes	22.8 (SD 9.2)	.910
No	23.0 (SD 9.1)	

Further analysis highlighted patients purchasing medications OOP had a higher symptom burden. Specifically, patients purchasing at least one OOP drug had a higher mean PERSONS score compared to those who did not [30.4 (SD 13.2) vs 17.6 (SD 12.3), *P* < .001]. This data was confirmed also in patients purchasing 3 or more OOP drugs [mean 35.9 (SD 11.3) vs 24.9 (SD 13.9), *P* < .001]. Feelings of financial instability were more common among patients who used more OOP drugs. Patients purchasing at least one OOP drug had significantly lower mean COST scores compared to those who did not [mean 21.3 (SD 8.9) vs 28.7 (SD 7.2), *P* < .001]. Similarly, those purchasing 3 or more OOP drugs had lower COST scores than those who did not [mean 19.3 (SD 8.7) vs 24.13 (SD 8.8), *P* < .001] (Table 5).

**Table 5.** Differences in COST and PERSONS in patients treated with or without ≥ OOP medication.

	COST		PERSONS	
	Mean (SD)	*P*-value	Mean (SD)	*P*-value
**At least 1 drug OOP**				
Yes	21.3 (SD 8.9)	<.001	30.4 (SD 13.2)	<.001
No	28.7 (SD 7.2)		17.6 (SD 12.3)	
**At least 3 drugs out of pocket**				
Yes	19.3 (SD 8.7)	<.001	35.9 (SD 11.3)	<.001
No	24.1 (SD 8.8)		24.9 (SD 13.9)	

Additionally, we observed that a higher symptom burden (PERSONS score) correlated with increased OOP medication use (*r* = 0.449, *P* < .001) and was inversely associated with financial toxicity (*r* = −0.431; *P* < .001), suggesting that greater symptom burden is linked to higher financial distress.

To further explore the relationship between symptom burden, financial toxicity and OOP medication use, we compared patients receiving supportive care with those who did not, using supportive care as a proxy for high symptom burden. Patients receiving supportive care (as simultaneous care during active treatment or as palliative care) had a higher PERSONS score compared to patients who did not [mean 31.9 (SD 11.7) vs 23.4 (SD 14.9), *P* < .001]. However, they also had greater financial instability, as reflected by lower COST score [21 (SD 7.7) vs 24.8 (SD 9.9), *P* < .002] and purchased more OOP medications [2.1 (SD 1.1) vs 1.2 (SD 1.2), *P* < .001].

These findings were confirmed and expanded among the 111 patients in active treatment, 37 (33.3%) of whom needed simultaneous care whereas 74 (66.7%) did not. In this subgroup, patients receiving simultaneous care displayed significantly higher mean PERSONS scores [30.1 (SD 10.8) vs 20.9 (SD 13.7), *P* < .001] along with a lower mean COST score [18.8 (SD 8.5) vs 26.2 (SD 9.9), *P* < .001] and a higher mean number of OOP drugs [2.2 (SD 1) vs 1.1 (SD 1.2), *P* < .001] compared to patients who did not receive simultaneous care (Table 6).

**Table 6.** Differences in COST and PERSONS in treated patients with or without simultaneous care.

	COST		PERSONS		OOP drugs	
	Mean ± SD	*P*-value	Mean ± SD	*P*-value	Mean ± SD	*P*-value
**Supportive care in whole patients population**						
Yes	21 ± 7.7	.002	31.9 ± 11.7	<.001	2.1 ± 1.1	<.001
No	24.8 ± 9.9		23.4 ± 14.9		1.2 ± 1.2	
**Supportive care in treated patients population**						
Yes	18.8 ± 8.5	<.001	30.1 ± 10.8	<.001	2.2 ± 1	<.001
No	26.2 ± 9.9		20.9 ± 13.7		1.1 ± 1.2	
**Supportive care in palliative setting**						
Yes	22.6 ± 9.8	.9	33.10 ± 12.3	.065	2.05 ± 1.2	.003
No	22.4 ± 6.7		27.80 ± 15.9		1.32 ± 1.2	

## Discussion

Our study examined the interaction between financial toxicity, symptom management, and well-being in patients with cancer. The key finding of our analysis is that the severity of symptoms and their management with OOP medications were significantly associated with financial toxicity and symptom burden. We observed a significant relationship between PERSONS scores and OOP medication usage, suggesting that patients with a higher symptom burden might incur greater medication expenses, leading to increased financial toxicity. This observation aligns with existing literature, which highlights that the costs of cancer-related symptom medications contribute substantially to the financial burden on patients with cancer. Patients often face expenses for medications not fully covered by insurance, including copayments, deductibles, and nonformulary prescription drugs. Consequently, the need for symptom management medications can exacerbate financial distress, particularly for patients with limited financial resources or insufficient insurance coverage.

Financial toxicity has been widely studied in the United States, where high healthcare costs often lead to catastrophic financial consequences, including medical debt and bankruptcy. The Italian healthcare system, in contrast, offers universal coverage through the Servizio Sanitario Nazionale (SSN), which provides cancer treatments free of charge.

However, previous Italian studies highlighted how the cost of medications for managing cancer-related symptoms can contribute substantially to the financial burden experienced by patients, despite Italy’s universal healthcare system.^22^

For example, Sparano et al.^23^ demonstrated that a significant portion of cancer patients in Italy report financial distress due to the cost of nonreimbursed supportive care medications and other OOP healthcare expenses.

Our findings confirm the observation that Italian patients still experience financial distress due to OOP expenses for supportive care, medications, and transportation costs. In the United States, the burden of healthcare costs is primarily borne by private insurance or OOP payments, leading to significantly higher rates of financial toxicity. Studies indicate that 42% of US cancer patients deplete their savings within 2 years of diagnosis.^24^ By contrast, Italy’s public healthcare system mitigates some of these effects, yet gaps remain in noncovered supportive care drugs and informal caregiving costs, which disproportionately affect lower-income patients.^25^

Prior Italian research has also shown that these gaps in coverage can lead to financial distress for cancer patients. A study by Lillini et al.^25^found that despite universal healthcare, 50% of Italian cancer patients reported significant financial hardship due to expenses for symptom management, transportation, and noncovered medications. Furthermore, Italian studies have emphasized the importance of regional disparities in healthcare access and financial support, which can exacerbate financial toxicity, especially in certain geographical areas.^22^

This geographical variability is critical, as it suggests that financial toxicity in Italy is not solely a function of healthcare system design but is also influenced by local policy implementation. However, our patient sample included participants from only a limited number of Italian regions, which restricted our ability to validate and generalize findings related to geographic disparities in financial toxicity across the broader Italian context. This limitation highlights the need for future research to adopt regionally representative sampling frameworks to more accurately capture the extent and underlying determinants of territorial variability in the financial burden among cancer patients. Several studies have shown the association between financial distress and symptom burden in patients with cancer. Research by Zafar et al.^10^ found that patients with cancer experiencing greater symptom severity report higher levels of financial distress and poorer quality of life.^26^ Similarly, Yabroff et al.^27^ reported that patients with multiple symptoms are more likely to incur high OOP healthcare costs, leading to an increased financial burden. The relationship between symptom burden and financial toxicity likely reflects a complex, bidirectional interplay. On the one hand, individuals with more severe or numerous symptoms may necessitate extensive pharmacologic and supportive interventions, often leading to increased OOP expenditures and subsequent financial strain. On the other hand, financial hardship itself may adversely affect patients’ capacity to access timely and appropriate symptom control, thereby intensifying their clinical burden through mechanisms such as psychological distress, reduced treatment adherence, and limited availability of supportive services. This cyclical interaction underscores the need for integrated approaches that address both clinical and economic dimensions of cancer care. Our findings suggest that financial toxicity in our patient population is higher than what has been reported in other studies. In 2 previous studies involving gynecologic cancer patients, the mean COST score was 30.0 (SD 9.46), with 23% of patients reporting financial difficulties, and 31.9 (IQR: 21-38), with 35% scoring below 26.^18,19^ The higher financial toxicity observed in our study could be related to the characteristics of our enrolled population, possibly due to their advanced disease stage and the high proportion of patients receiving palliative care.

A crucial finding from our study is that financial distress correlates with higher symptom burden and lower treatment adherence, a trend also observed in the United States. Patients experiencing financial hardship may reduce medication use, skip doses, or forgo supportive care services. Italian patients reported similar behaviors, particularly for symptom management drugs not covered by the SSN. This suggests that while Italy’s system reduces the impact of direct treatment costs, indirect financial barriers persist, mirroring US challenges. Our study identified lower educational attainment and unemployment as risk factors for financial toxicity in Italy, consistent with US findings. Educational level influences health literacy and the ability to navigate financial assistance programs, both in Italy and the United States. Unlike the United States, where charity care and pharmaceutical assistance programs play a larger role, Italy’s reliance on regional reimbursement schemes creates disparities in financial support availability.

Given these findings, financial toxicity should be considered a global issue, even in high-income nations, and policy measures are needed to address it, as this problem is increasingly recognized worldwide.

In conclusion, our findings underscore the substantial financial burdens borne by Italian cancer patients, particularly those reliant on OOP interventions for symptom relief. While the NHS offsets much of the direct expenditure associated with oncologic care, unaddressed gaps in coverage perpetuate significant economic strain—mirroring patterns observed in the United States and other high-income nations that employ decentralized healthcare mechanisms.

Future investigations should examine the longitudinal ramifications of financial toxicity in diverse healthcare environments, prioritizing policy reforms that strengthen financial protections and broaden access to supportive services. Cross-national comparative analyses will yield crucial insights for designing targeted interventions that mitigate economic hardship and ultimately enhance patient outcomes.

## Supplementary Material

oyaf131_suppl_Supplementary_Material_1

oyaf131_suppl_Supplementary_Material_2

oyaf131_suppl_Supplementary_Material_3

oyaf131_suppl_Supplementary_Table_S1

## Data Availability

Raw data will be provided upon reasonable request.
