# Supplementary material for: The financial toll of cancer: uncovering the links between financial toxicity and symptom burden
Source: Oncologist. 2025 Jun 26;30(6):oyaf131. doi: 10.1093/oncolo/oyaf131 (PMC12199244; doi:10.1093/oncolo/oyaf131)
Supplement: oyaf131_suppl_Supplementary_Material_2 [file oyaf131_suppl_supplementary_material_2.docx]

Below is a list of statements that other people with your illness have said are important. **Please circle or mark one number per line to indicate your response as it applies to the past 7 days.**

|  |  | **Not at all** | | **A little bit** | **Somewhat** | **Quite a bit** | **Very much** |
| --- | --- | --- | --- | --- | --- | --- | --- |
|  |  |  |  |  |  |  |  |
| FT1 | I know that I have enough money in savings, retirement, or assets to cover the costs of my treatment | | 0 | 1 | 2 | 3 | 4 |
| FT2 | My out-of-pocket medical expenses are more than I thought they would be | | 0 | 1 | 2 | 3 | 4 |
| FT3 | I worry about the financial problems I will have in the future as a result of my illness or treatment | | 0 | 1 | 2 | 3 | 4 |
| FT4 | I feel I have no choice about the amount of money I spend on care | | 0 | 1 | 2 | 3 | 4 |
| FT5 | I am frustrated that I cannot work or contribute as much as I usually do | | 0 | 1 | 2 | 3 | 4 |
| FT6 | I am satisfied with my current financial situation | | 0 | 1 | 2 | 3 | 4 |
| FT7 | I am able to meet my monthly expenses | | 0 | 1 | 2 | 3 | 4 |
| FT8 | I feel financially stressed | | 0 | 1 | 2 | 3 | 4 |
| FT9 | I am concerned about keeping my job and income, including paid work at home | | 0 | 1 | 2 | 3 | 4 |
| FT10 | My cancer or treatment has reduced my satisfaction with my present financial situation | | 0 | 1 | 2 | 3 | 4 |
| FT11 | I feel in control of my financial situation | | 0 | 1 | 2 | 3 | 4 |
| FT12 | My illness has been a financial hardship to my family and me | | 0 | 1 | 2 | 3 | 4 |
