# Supplementary material for: The financial toll of cancer: uncovering the links between financial toxicity and symptom burden
Source: Oncologist. 2025 Jun 26;30(6):oyaf131. doi: 10.1093/oncolo/oyaf131 (PMC12199244; doi:10.1093/oncolo/oyaf131)
Supplement: oyaf131_suppl_Supplementary_Material_3 [file oyaf131_suppl_supplementary_material_3.docx]

| **Pain** | 0 | 1 | 2 | 3 | 4 | 5 | 6 | 7 | 8 | 9 | 10 |
| --- | --- | --- | --- | --- | --- | --- | --- | --- | --- | --- | --- |
| **Eating** | 0 | 1 | 2 | 3 | 4 | 5 | 6 | 7 | 8 | 9 | 10 |
| **Rehabilitation** | 0 | 1 | 2 | 3 | 4 | 5 | 6 | 7 | 8 | 9 | 10 |
| **Sleep** | 0 | 1 | 2 | 3 | 4 | 5 | 6 | 7 | 8 | 9 | 10 |
| **Oxygen** | 0 | 1 | 2 | 3 | 4 | 5 | 6 | 7 | 8 | 9 | 10 |
| **Nausea/Vomiting** | 0 | 1 | 2 | 3 | 4 | 5 | 6 | 7 | 8 | 9 | 10 |
| **Suffering** | 0 | 1 | 2 | 3 | 4 | 5 | 6 | 7 | 8 | 9 | 10 |

**The PERSONS score**
