# Supplementary material for: The financial toll of cancer: uncovering the links between financial toxicity and symptom burden
Source: Oncologist. 2025 Jun 26;30(6):oyaf131. doi: 10.1093/oncolo/oyaf131 (PMC12199244; doi:10.1093/oncolo/oyaf131)
Supplement: oyaf131_suppl_Supplementary_Table_S1 [file oyaf131_suppl_supplementary_table_s1.docx]

Table S1a - Out of pocket medication

|  | N. (%) |
| --- | --- |
| At least 1 drug out of pocket | 163 (77.3%) |
| At least 3 drugs out of pocket | 50 (23.7%) |
| None | 48 (22.7%) |
|  |  |
| Out of pocket medication |  |
| Pain relief | 95 (45%) |
| Anti-Diarrheal | 23 (10.9%) |
| Constipation | 59 (28%) |
| Anti-emetics | 37 (17.5%) |
| Anorexia and Cachexia | 20 (9.5%) |
| Cancer-Associated Fatigue | 44 (20.9%) |
| Mood and anxiety | 17 (8.1%) |
| Food supplements | 29 (13.7%) |
| Mobility aids, nursing care beds and others assistive devices | 25 (11.8%) |

Table S1b. PERSONS scores. Values are expressed as mean and standard deviation

| PERSONS symptoms | Assessment |
| --- | --- |
| **P: pain** | 3.2±2.8 |
| **E: Eating** | 3.3±3.0 |
| **R: Rehabilitation** | 5.4±3.0 |
| **S: Sleeping disorder** | 3.2±3.2 |
| **O: O2 Dyspnea** | 1.7±2.6 |
| **N: Nausea/emesis** | 1.2±2.3 |
| **S: Suffering** | 4.7±3.1 |
| **Total score** | 3.3±3.2 |
